# Effect of an Acute Prolonged Overnight Fast on Skeletal Muscle Insulin and Anabolic Sensitivity in Healthy Men

**DOI:** 10.1016/j.tjnut.2026.101660

**Published:** 2026-06-07

**Authors:** Joanne E Mallinson, Mia Keeton, Aline Nixon, Abhishek Sheth, Robert Jones, Pardeep Pabla, Joan MG Senden, Luc JC van Loon, Kostas Tsintzas

**Affiliations:** 1 School of Life Sciences, Division of Physiology, Pharmacology and Neuroscience, University of Nottingham Medical School, Queen’s Medical Centre, Nottingham, United Kingdom; 2 Department of Human Biology, NUTRIM Institute for Nutrition and Translational Research in Metabolism, Maastricht University, Maastricht, The Netherlands

**Keywords:** fasting, skeletal muscle, protein metabolism, anabolic sensitivity, insulin sensitivity

## Abstract

**Background:**

Repeated prolonged overnight fasting enhances anabolic sensitivity and preserves myofibrillar protein synthesis. Whether a single fasting episode can enhance dietary protein-derived amino acid incorporation into skeletal muscle protein remains unclear.

**Objective:**

To investigate the effect of a single 16-h compared with 10-h overnight fast on postprandial insulin and anabolic sensitivity in humans.

**Methods:**

Randomized, crossover study. On 2 occasions, healthy participants (*n* = 9; age 22.6 ± 3.5 y; body mass index: 24.0 ± 2.5 kg/m^2^; homeostasis model assessment of insulin resistance 1.00 ± 0.54) ingested a liquid meal, which consisted of specifically produced, intrinsically [1-^13^C]phenylalanine–labeled milk protein (0.35 g/kg body mass, bm) and dextrose (0.8 g/kg bm), dissolved in 6 mL/kg bm water, after either a 10-h (Control) or 16-h (prolonged overnight fast trial, Long OF) overnight fast. Skeletal muscle and blood samples were obtained before, during, and 3 h after meal ingestion.

**Results:**

Postprandial incorporation of dietary protein-derived [1-^13^C]phenylalanine into myofibrillar protein did not differ between Control and Long OF (0.018 ± 0.005 compared with 0.022 ± 0.005 mole percent excess, *d*_s_ = 0.22, *P* = 0.56). Forearm branched amino acid uptake incremental area under the curve [D (95% confidence interval, CI) 103 (−1618, 1823) μmol/min^.^180 min, *d*_s_ = 0.05, *P* = 0.892] and glucose uptake [D (95% CI) 24.7 (−25.3, 74.8) μmol/min, *η*^2^*p* = 0.16, *P* = 0.280] also did not differ between treatments, but the postprandial serum insulin response [D (95% CI) −7.4 (−14.4, −0.4) mIU/L, *η*^2^*p* = 0.42, *P* = 0.042] was lower in Long OF.

**Conclusions:**

A single prolonged overnight fast does not enhance postprandial skeletal muscle insulin sensitivity or dietary protein-derived amino acid incorporation into skeletal muscle protein in healthy, young adults. Whether susceptible populations, such as older, insulin-resistant individuals, may benefit from a more prolonged overnight fast warrants investigation.

This study was registered at clinicaltrials.gov as NCT05420181.

## Introduction

Time-restricted eating (TRE), a form of intermittent fasting that confines daily energy intake to a restricted window (typically 6–10 h), extends the duration of the overnight fast [[1], [2], [3]]. TRE has been shown to confer multiple cardiometabolic benefits independent of weight loss, both in healthy individuals [4] and in those with obesity or impaired glucose tolerance [5]. However, there is evidence suggesting that TRE, particularly when associated with ad libitum food intake, may lead to a greater reduction in fat-free mass [6,7]. This may reflect an unintentional reduction in energy and protein intake, potentially reducing muscle protein synthesis (MPS) and lean tissue preservation compared with equivalent energy restriction without fasting [8]. Notably, short-term (10 d) TRE in individuals with obesity does not appear to reduce daily myofibrillar protein synthesis rates, despite modest reductions in total, but not appendicular, lean mass [9].

We previously demonstrated that a 2-wk early TRE intervention (8 h feeding from 08:00 to 16:00) improves whole-body and skeletal muscle insulin sensitivity in healthy individuals [4]. Although changes in body and lean mass were small, skeletal muscle uptake of branched-chain amino acids (BCAAs) across the forearm was greater after TRE compared with habitual eating (∼13 h of feeding from 08:00 to 21:00). However, the metabolic fate of these BCAAs remains uncertain, as incorporation into muscle protein, an index of protein synthesis, was not assessed. Moreover, because postintervention assessments were performed after a standardized overnight fast (∼10 h) in both conditions, it is unclear whether a single episode of prolonged overnight fast (∼16 h), independent of chronic dietary changes or weight and lean mass losses, can acutely enhance anabolic and insulin sensitivity and stimulate MPS in response to dietary protein.

Therefore, this study examined the effect of a single 16-h compared with 10-h overnight fast on postprandial metabolism, insulin sensitivity, and skeletal muscle anabolic sensitivity in healthy humans. It was hypothesized that extending the overnight fasting duration to 16 h would preserve anabolic sensitivity and even augment the capacity for postprandial incorporation of dietary protein-derived amino acids in skeletal muscle protein in healthy adults.

## Methods

### Participants

Ten healthy men, aged 18–35 y, with a BMI of ≤27 kg/m^2^, were recruited to the trial under protocols approved by the University of Nottingham Faculty of Medicine and Health Sciences Research Ethics Committee (Reference No FMHS 289-1904)*.* Individuals were excluded if they regularly skipped breakfast; were on an energy-restricted diet and/or experienced significant weight fluctuation in the previous 3 mo (>5% body weight); had a self-reported score of >20 (a marker of symptoms and concerns characteristic of eating disorders) on the Eating Attitudes Test (EAT-26) [10]; had active metabolic, endocrine, or cardiovascular abnormalities, including hypertension or heart failure. One individual was excluded for not meeting the inclusion criteria.

### Experimental design

This was a single-center, randomized, 2-arm, crossover study (clinicaltrials.gov Identifier NCT05420181) that took place in the David Greenfield Human Physiology Unit at the University of Nottingham. After written and signed informed consent, eligible participants attended the laboratory on 4 occasions: 1 medical screening session, 1 preliminary session to assess their resting metabolic rate, and 2 main experimental visits, ≥1 wk apart.

During the medical screening, all participants completed a medical history, physical activity (International Physical Activity Questionnaire (IPAQ)) [11] and EAT-26 questionnaires. They also undertook 12-lead electrocardiogram, blood pressure, weight, and height measurements, and had a blood sample taken for routine chemistry and hematology investigation. Each participant’s screening information was reviewed and approved by a medical doctor before entering the study. Once eligibility was confirmed, all participants were instructed to complete a 3-d food diary for the assessment of their habitual dietary and physical activity patterns, along with the temporal distribution of energy intake throughout the day. On a separate preliminary visit to the laboratory, all participants had their resting energy expenditure (REE) assessed by indirect calorimetry. Using REE and individual daily physical activity level allowed for the estimation of their daily energy requirements.

Eligible participants were then randomly divided using computer-generated randomization software (https://www.randomizer.org) to undergo 2 experimental trials that involved the ingestion of a standardized oral liquid meal after 2 different durations (10 h compared with 16 h) of overnight fast. On each occasion, participants ingested a bolus of milk protein (0.35 g/kg body mass, bm), which was intrinsically labeled with [1-^13^C]phenylalanine, along with 0.8 g/kg bm of dextrose and 2 g of chocolate powder [containing 0.46 g protein, 0.2 g carbohydrate (CHO), 0.4 g fat] dissolved in 6 mL/kg bm of water.

### Experimental protocol

On each main intervention trial, participants reported to the laboratory at 08:00, after an overnight fast (10 or 16 h), having abstained from intense exercise and alcohol consumption for the previous 72 h and caffeine consumption on the day of the visit. At least 48 h before each trial, participants were fitted with a subcutaneous continuous glucose monitoring (CGM) device (FreeStyle Libre, Abbott Diabetes Care), which measured interstitial glucose concentrations every 15 min throughout the entire 24-h period leading to their intervention visit. Participants were provided with customized food menus, that were based on their estimated isoenergetic daily energy requirements, to consume the day before each visit. On both occasions, the total daily energy intake was 12,528 ± 2111 (mean ± SD) kJ, with 33.0% ± 1.4% of energy obtained from fat, 51.4% ± 1.7% from CHO, and 15.7% ± 0.9% from protein. Furthermore, all participants consumed a standardized evening meal provided by the investigators at either 16:30 (prolonged overnight fast trial; Long OF) or 22:30 (Control trial) the day before the main trial. The evening meal provided 47.5% of energy from CHO, 34.0% from fat, and 18.5% from protein.

Participants were asked to consume this evening meal within 30 min and report to the unit at 08:00 the next day (with no food or drinks other than water allowed during the intervening period). Upon arrival, participants rested in a semisupine position in a hospital bed for 30 min. After this, baseline REE and whole-body substrate oxidation rates were determined for 20 min via indirect calorimetry using a flow-based-dilution canopy hood (Quark RMR, Cosmed, IT). A cannula was inserted retrograde into a superficial dorsal vein of the dominant hand, which was kept in a warming unit (55°C) to arterialize the venous drainage [12]. A second cannula was inserted into a deep antecubital vein of the other arm using ultrasound guidance for sampling of deep venous blood. Both cannulae were kept patent via a saline drip. Immediately after this, baseline blood samples were simultaneously obtained from each cannula along with measurements of blood flow of the brachial artery using Doppler ultrasound. Differences between arterialized venous (from the hand) and deep venous (from the antecubital vein) glucose concentrations, along with measurements of blood flow of the brachial artery (*BF*_*BA*_; expressed in mL/min) using Doppler ultrasound, were used to determine rates of glucose (G) flux (expressed in μmol/min) across the forearm using the following equation: *G*_*flux*_ = ([*G*]_*arterialized*_ − [*G*]_*venous*_) × *BF*_*BA*_. This provided an index of local muscle insulin sensitivity. Similarly, the flux of BCAA (expressed in μmol/min) across the forearm provided an index of local muscle anabolic sensitivity. After this, a muscle biopsy sample was obtained from the vastus lateralis muscle of 1 randomly assigned leg using the percutaneous needle biopsy technique as described previously [13]. Muscle biopsies were rapidly frozen and stored in liquid nitrogen for subsequent analysis.

At 09:00, either 10 or 16 h after the last meal, the participant was provided with an oral liquid meal consisting of dextrose and the intrinsically labeled milk protein as described above. They were asked to consume this meal within 10 min. During the subsequent 3-h postprandial period, blood samples were obtained from each cannula every 15 min for the analysis of blood metabolite and hormone concentrations as described below. Simultaneous measurements of blood flow of the brachial artery were also obtained at each sampling point. Indirect calorimetry measurements were made every hour, and a second muscle biopsy sample from the vastus lateralis was obtained at 3 h after ingestion of the test meal.

### Blood analysis

Whole blood glucose concentrations were determined using a Yellow Springs Instrument Analyzer (YSI, 2300 STAT PLUS). Serum was separated from 1 aliquot of blood by centrifugation (15 min at 3000 × *g*) after being allowed to clot and analyzed for insulin concentrations by radioimmunoassay (EZHI-14K, Merk Millipore). Another aliquot of blood was collected into a tube containing 30 μL EGTA glutathione and centrifuged immediately at 3000 × *g* for 15 min at 4°C to obtain plasma that was aliquoted into a tube containing tetrahydrolipostatin (30 μg/mL of plasma) for the determination of free fatty acid (FFA) using a commercially available kit (NEFA HR-2, Wako). A separate aliquot of blood was transferred to an EDTA tube containing dipeptidyl peptidase-4 inhibitor (10 μL/mL plasma) for the determination of total gastric inhibitory polypeptide (GIP) by enzyme-linked immunoassay (EZHGIP-54K; Merck Millipore). Plasma BCAA (leucine, isoleucine, and valine) were analyzed using hydrophilic interaction liquid chromatography coupled to high-resolution MS as previously described [14]. After deproteinization on ice with dry 5-sulfosalicylic acid, another aliquot of plasma separated from EGTA-treated blood was also analyzed for phenylalanine concentrations, and [1-^13^C]phenylalanine enrichments by GC-MS (Agilent 7890A GC/5975C; MSD) after derivatization with tert-butyl dimethylsilyl as previously described [15,16].

### Skeletal muscle analysis

Myofibrillar protein–enriched fractions were extracted from 50 mg of wet muscle tissue by hand-homogenizing on ice using a pestle in a standard extraction buffer (7 μL/mg). The samples were centrifuged at 700 × *g* and 4°C for 15 min. The pellet was washed with 500 μL double-distilled H_2_O and centrifuged at 700 × *g* and 4°C for 10 min. The myofibrillar protein was solubilized by adding 1 mL of 0.3 M NaOH and heating at 50°C for 30 min with vortex mixing every 10 min. Samples were centrifuged at 9500 × *g* and 4°C for 5 min, and the supernatant containing the myofibrillar proteins was collected. The remaining pellet was washed with 1 mL of 0.3 M NaOH and then centrifuged at 9500 × *g* and 4°C for 5 min. Subsequently, the supernatant was collected and added to the first supernatant. The remaining collagen pellet was discarded. Myofibrillar proteins were precipitated by the addition of 1 mL of 1 M perchloric acid and spinning at 700 × *g* and 4°C for 10 min. The myofibrillar protein was washed twice with 70% ethanol and hydrolyzed overnight in 2 mL of 6 M HCL at 110°C. The free amino acids from the hydrolyzed myofibrillar protein pellet were dried under a nitrogen stream at the same time as being heated to 110°C. The free amino acids were then dissolved in 25% acetic acid solution, passed over cation exchange AG 50W-X8 resin columns (mesh size: 100–200, ionic form: hydrogen; Bio-Rad Laboratories), and eluted with 2 M NH4OH. To determine myofibrillar protein L-[1-^13^C]-phenylalanine enrichments by GC-C-IRMS analysis, the purified amino acids were converted into N-ethoxycarbonyl ethyl ester derivatives with ethyl chloroformate. The derivatives were then measured by GC-C-IRMS (MAT 253; Finnigan) using a DB5-MS-column (no. 122–5532; Agilent J+W Scientific GC Column, GC Isolink; Agilent Technologies) and monitoring of ion masses 44, 45, and 46.

### Outcomes

#### Primary endpoints

Indices of skeletal muscle anabolic sensitivity: BCAA (sum of leucine, isoleucine, and valine) uptake across the forearm (upon which the power calculation was based); [1-^13^C]phenylalanine incorporation into the myofibrillar protein pool. Indices of whole-body insulin sensitivity: Matsuda Index; postprandial blood glucose and insulin incremental areas under the curve (iAUC). Although multiple outcomes were registered as coprimary endpoints, the study was powered based on a single outcome, reflecting its role as the principal inferential outcome. Primary endpoints were tested in the order they appear above.

#### Secondary endpoints

Glucose uptake across the forearm as an index of skeletal muscle insulin sensitivity; whole body CHO and fat oxidation rates; circulating FFA and GIP responses. Sample size estimations were completed using G∗Power software v3.1.9.7. Based on previous observations [4], the analysis indicated that a total of 9 participants were required to detect a difference of 1.25 (SD 0.89) nmol/min/kg in forearm muscle BCAA uptake between the 2 experimental arms with a power of 90% at 5% significance level (2-tail test).

### Data handling and statistical analysis

Food diaries were kept by participants for 3 full days during the baseline period. Dietary analysis was performed using Nutritics (version 5.096; Nutritics Ltd). Daily energy intake (E) and CHO, fat, and protein content (in g and %E) of all meals and snacks were calculated. CGM was recorded for 1 full day before each treatment visit. Recording started ≥48 h before participants reported to the laboratory on the day of each main visit and terminated at 08:00 on the day of the visit. Only data for the last 24 h of the run-in period to the main trial were analyzed to avoid bias because of insertion of the sensor and allow sufficient stabilization of the CGM system. CGM data were shifted back to the nearest 15-min timepoint, and days with ≥20% missing data were excluded. The Coefficient of Variation (CV) for interstitial glucose concentrations, an index of glucose variability that allows comparisons between individuals with different mean glycemic values, was calculated as % using the formula: (SD/mean glucose) × 100.

Statistical analysis was performed in Prism V.10.3.0 (GraphPad). Descriptive data are presented as mean ± SD. Experimental data are presented as mean ± SEM and were tested for normality using the Shapiro–Wilk test. iAUC was calculated using the trapezoidal method. [1-^13^C]phenylalanine incorporation into the myofibrillar protein pool and iAUC data were analyzed using paired Student’s *t*-test. Cohen’s *d*_*s*_ was used to quantify the effect size for comparisons performed using paired *t*-tests. All outcomes with multiple timepoints were analyzed using a 2-way analysis of variance (ANOVA), with both factors (sampling time and experimental treatment) as within-subjects factors. Partial eta squared values (*η*^2^*p*) were calculated to report the effect size for treatment effects obtained from ANOVA. All resulting *P* values for post hoc multiple comparisons after ANOVA that showed significant main effects were adjusted using Tukey's tests. Statistical significance was accepted at *P* < 0.05. Comparisons of the mean differences between treatment groups are reported as *D* change scores with 95% confidence intervals (CI). The effect of treatment on outcome variables was deemed significant (*P* < 0.05) for means whose 95% CIs did not contain 0. The exact *P* values cited alongside effect sizes and 95% CIs are derived from either the treatment effect of the 2-way ANOVA, for variables with multiple timepoints, or from paired *t*-tests, for variables with a single timepoint.

## Results

### Participants

**FIGURE 1:**
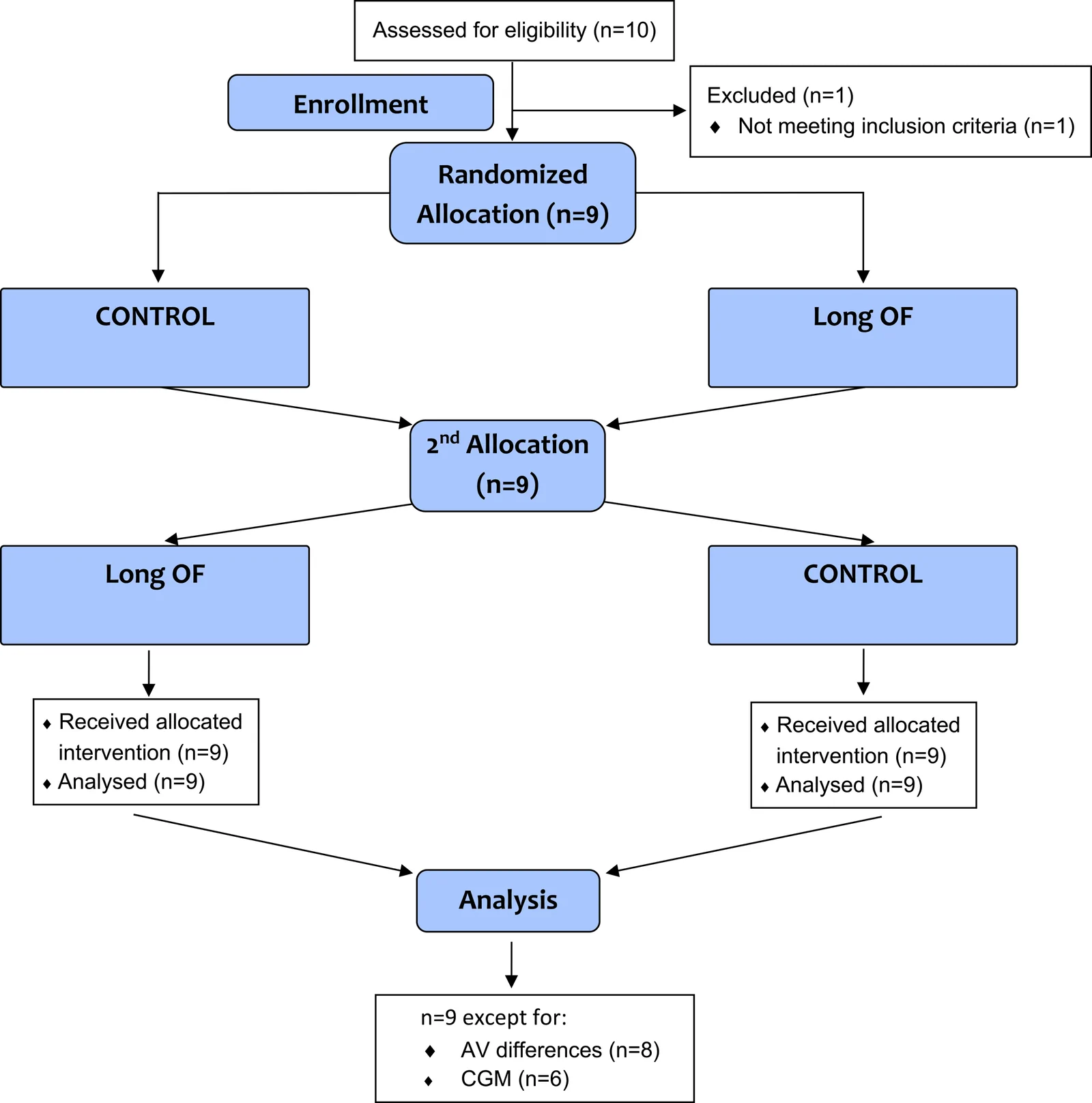
Study CONSORT flow diagram. AV, arterio-venous differences; CGM, continuous glucose monitoring; Long OF, prolonged overnight fast trial.

Ten physically active men were assessed for eligibility (Figure 1), of whom 9 were found eligible and randomly divided and treated between 30 May, 2019 and 19 March, 2024. All 9 participants completed the study. The participant baseline demographics are shown in Table 1.

**TABLE 1:** Participant characteristics

	(*n* = 9)
Age (y)	22.6 ± 3.5
Sex	All men
Height (m)	1.78 ± 0.07
Weight (kg)	75.9 ± 8.2
BMI (kg/m^2^)	24.0 ± 2.5
HOMA-IR	1.00 ± 0.54

Values are means ± SD. A surrogate index of insulin resistance (fasting blood glucose × fasting blood insulin/22.5).

### Plasma phenylalanine

**FIGURE 2:**
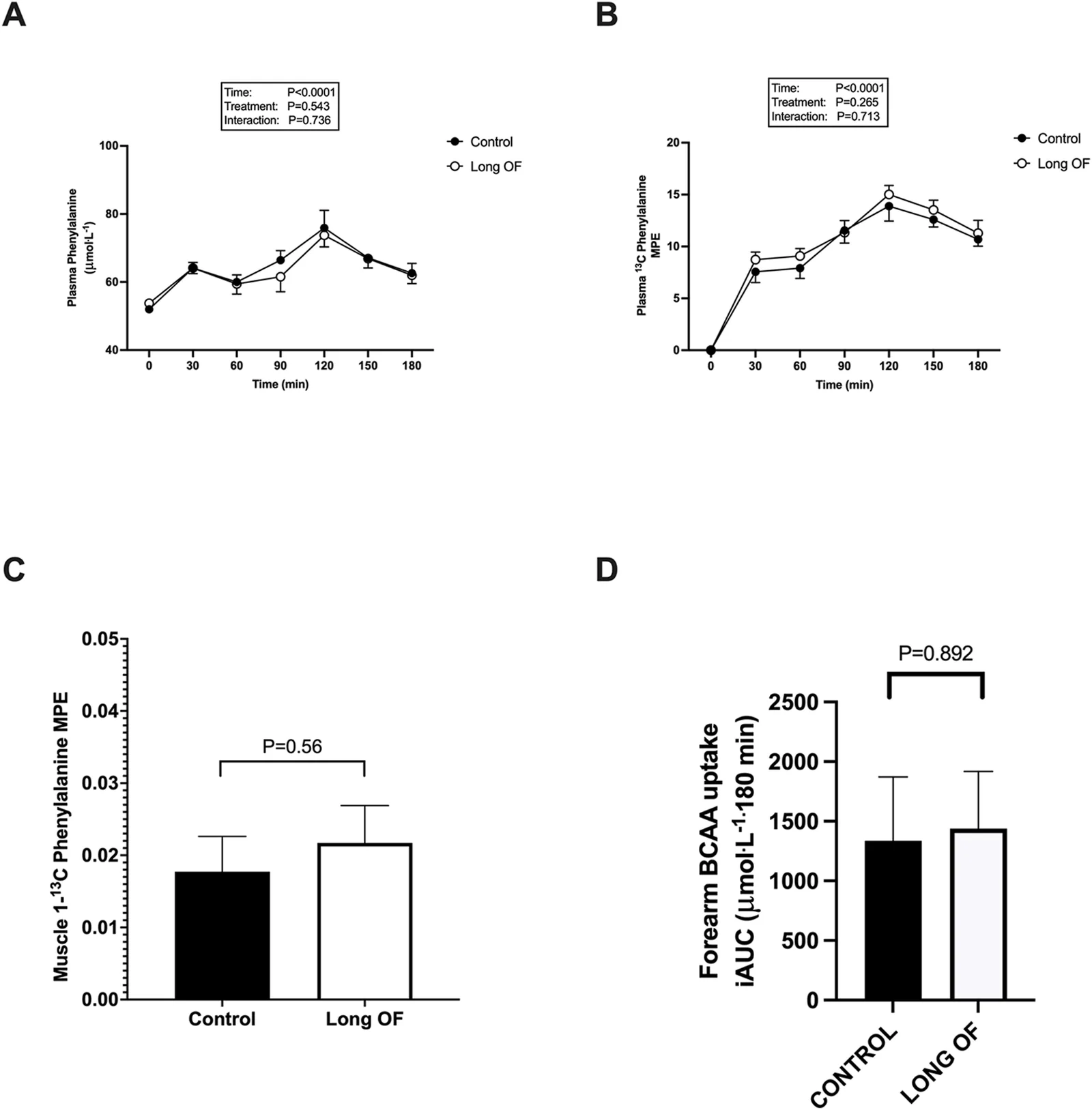
Plasma amino acid responses, plasma enrichment of [1-^13^C]phenylalanine, and incorporation into muscle protein. Effects of 16-h (Long OF) vs. 10-h (Control) overnight fast on (A) plasma enrichment of [1-^13^C]phenylalanine (MPE); (B) plasma phenylalanine concentrations; (C) skeletal muscle [1-^13^C]phenylalanine MPE; and (D) forearm BCAA uptake iAUC (*n* = 8). Values are means ± SEM; *n* = 9 except when indicated otherwise. *P* values for the main effects of 2-way analysis of variance are displayed in text boxes. *P* values for comparisons of the mean differences between treatments are also reported. BCAA, branched-chain amino acid; iAUC, incremental area under the curve; Long OF, prolonged overnight fast trial; MPE, mole percent excess.

Plasma phenylalanine (Figure 2A) concentrations increased in both trials after the ingestion of the milk protein (time effect *P* < 0.0001) with no significant differences between treatments (treatment effect *P* = 0.543).

### Plasma and muscle tissue protein [1-^13^C]phenylalanine enrichments

Plasma [1-^13^C]phenylalanine enrichment [mole percent excess (MPE)] increased after ingestion of the intrinsically labeled milk protein (time effect *P* < 0.0001) and reached a stable level after 120 min. No significant differences were observed between treatments [D (95% CI): −0.73 (−2.14, 0.68) MPE, *η*^2^*p* = 0.19; treatment effect *P* = 0.27] (Figure 2B). Skeletal muscle [1-^13^C]phenylalanine MPE, reflecting the incorporation of dietary protein-derived amino acids into the skeletal muscle myofibrillar protein pool, also did not differ between Control and Long OF (0.018 ± 0.005 compared with 0.022 ± 0.005 MPE, respectively) with D (95% CI) between treatments being −0.004 (−0.011, 0.019) MPE, *d*_*s*_ = 0.22, *P* = 0.56 (Figure 2C).

### Forearm BCAA uptake

Forearm BCAA uptake iAUC [D (95% CI): 103 (−1618, 1823) μmol/min^.^180 min, *d*_*s*_ = 0.05, *P* = 0.892] (Figure 2D) was not significantly different between Control and Long OF.

### Blood metabolic markers

**FIGURE 3:**
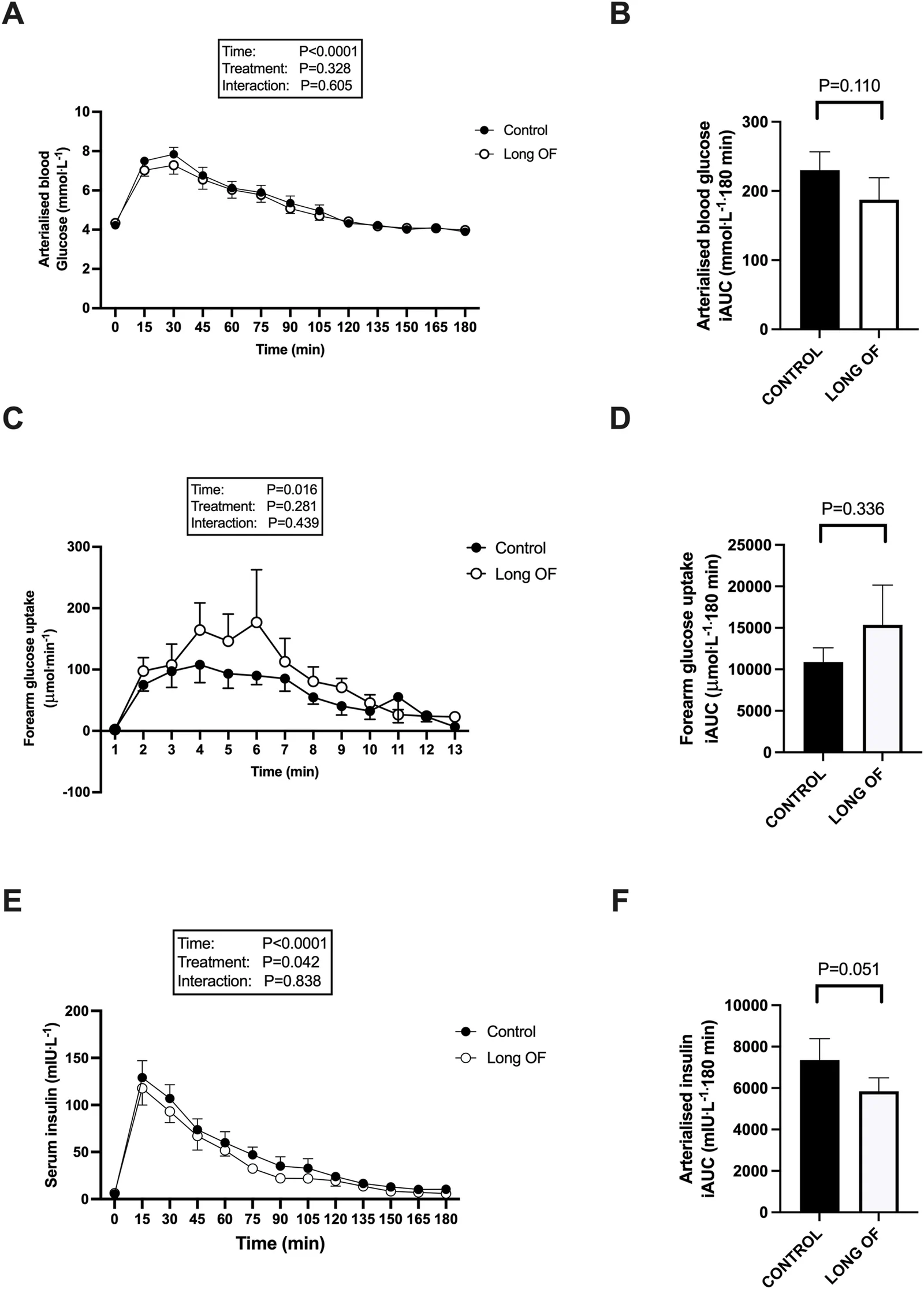
Blood metabolic responses. Effects of 16-h (Long OF) vs. 10-h (Control) overnight fast on (A) arterialized blood glucose concentrations; (B) arterialized blood glucose iAUC; (C) forearm glucose uptake over time (*n* = 8); (D) forearm glucose uptake iAUC (*n* = 8); (E) serum insulin concentrations; and (F) serum insulin iAUC. Values are means ± SEM; *n* = 9 except when indicated otherwise. *P* values for the main effects of 2-way analysis of variance are displayed in text boxes. *P* values for comparisons of the mean differences between treatments are also reported. iAUC, incremental area under the curve; Long OF, prolonged overnight fast trial.

Blood glucose concentrations were not significantly different between Control and Long OF (Figure 3A) [D (95% CI): 0.12 (−0.14, 0.38) mmol/L, *η*^2^*p* = 0.18; treatment effect *P* = 0.328]. Similarly, blood glucose iAUC was not significantly different between trials [D (95% CI): −34.7 (−83.2, 13.8) mmol/L^.^180 min, *d*_*s*_ = 0.60, *P* = 0.110] (Figure 3B).

Forearm glucose uptake [D (95% CI): 24.7 (−25.3, 74.8) μmol/min, *η*^2^*p* = 0.16, treatment effect *P* = 0.280] (Figure 3C) and iAUC [D (95% CI): 4475 (−5776, 14726) μmol/min^.^180min, *d*_*s*_ = 0.36, *P* = 0.336] (Figure 3D) were not significantly different between Control and Long OF.

Serum insulin concentrations increased in both trials, but the overall response was lower (treatment effect *P* = 0.042) in the Long OF than Control [D (95% CI): −7.4 (−14.4, −0.4) mIU/L, *η*^2^*p* = 0.42] (Figure 3E). As a result, serum insulin iAUC tended to be lower in Long OF than Control [D (95% CI): −1441 (−2896, 14) mIU/L^.^180 min, *d*_*s*_ = 0.75, *P* = 0.051] (Figure 3F).

### Whole-body insulin sensitivity

The Matsuda Index of whole-body insulin sensitivity was similar between Control and Long OF (8.79 ± 1.53 compared with 11.16 ± 2.31, respectively) with D between trials (95% CI) being 2.38 (−6.24, 1.48), *d*_*s*_ = 0.47, *P* = 0.193.

### Continuous glucose monitoring

**FIGURE 4:**
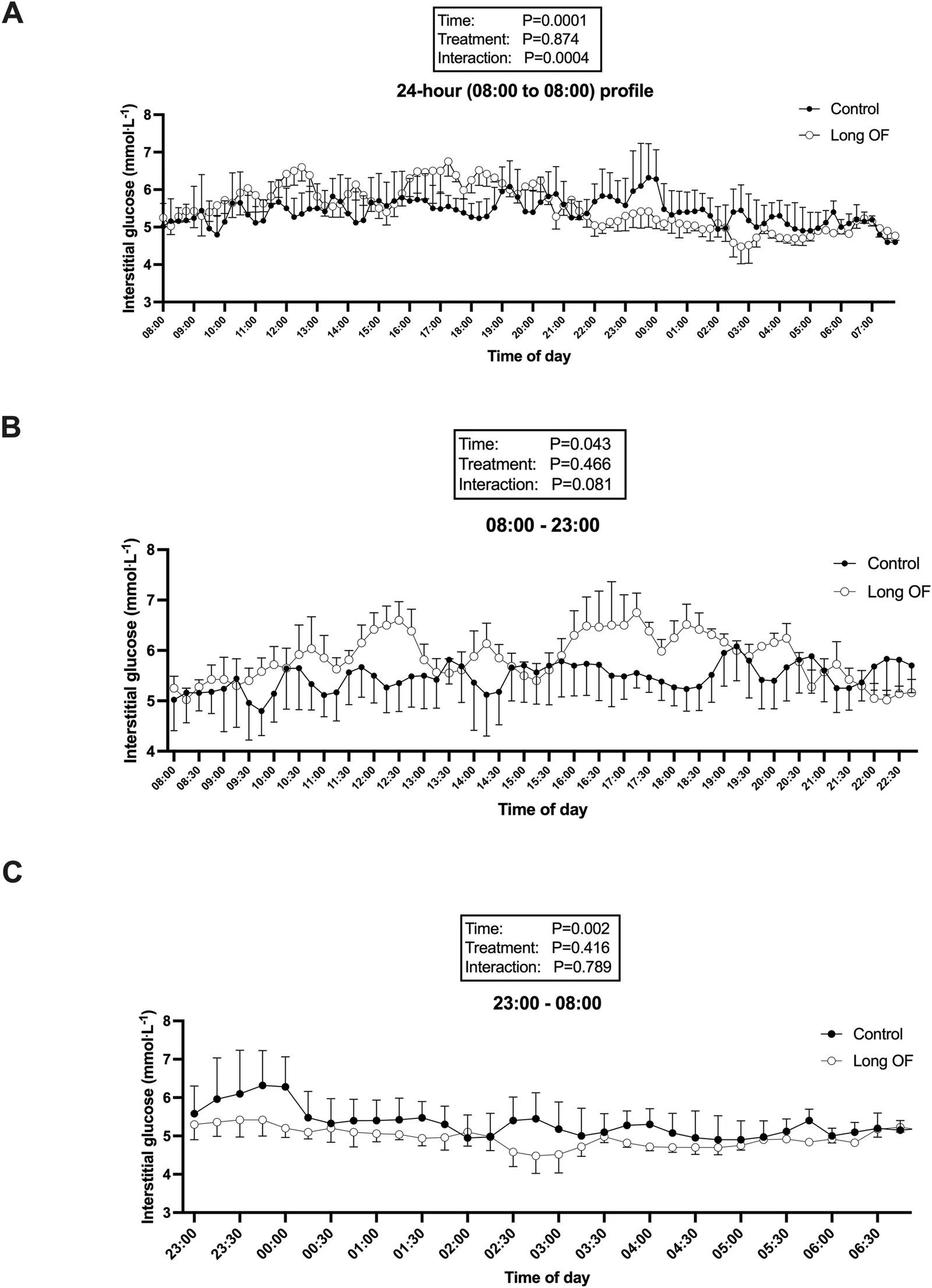
Continuous glucose monitoring (CGM). Effects of 16-h (Long OF) vs. 10-h (Control) overnight fast on (A) 24-h (08:00–20:00) interstitial glucose concentrations; (B) diurnal (08:00–23:00) interstitial glucose concentrations; and (C) nocturnal (23:00–08:00) interstitial glucose concentrations. Values are means ± SEM; *n* = 6. *P* values for the main effects of 2-way analysis of variance are displayed in text boxes. Long OF, prolonged overnight fast trial.

Interstitial glucose 24-h profiles for the main interventions are displayed in Figure 4A. Analysis of absolute glucose concentrations over 24 h (08:00–20:00) revealed a significant treatment by sampling time interaction (*P* = 0.0004). This was driven by a trend for higher diurnal (08:00–23:00; Figure 4B) glucose responses in Long OF (which was expected given that the same total food intake was consumed within a shorter eating window in the latter treatment) but numerically attenuated responses at night (23:00–08:00) when compared with Control (Figure 4C). As a result, there were no significant differences in mean 24 h glucose concentrations between interventions (Control 5.48 ± 0.44 mmol/L; Long OF 5.57 ± 0.14 mmol/L, *d*_*s*_ = 0.10, *P* = 0.818). Similarly, 24 h glycemic variability (%CV) was not significantly different between Control and Long OF (15.5% ± 1.7% compared with 15.0% ± 2.1%, *d*_*s*_ = 0.08, *P* = 0.846, respectively).

### Serum GIP and plasma FFA

**FIGURE 5:**
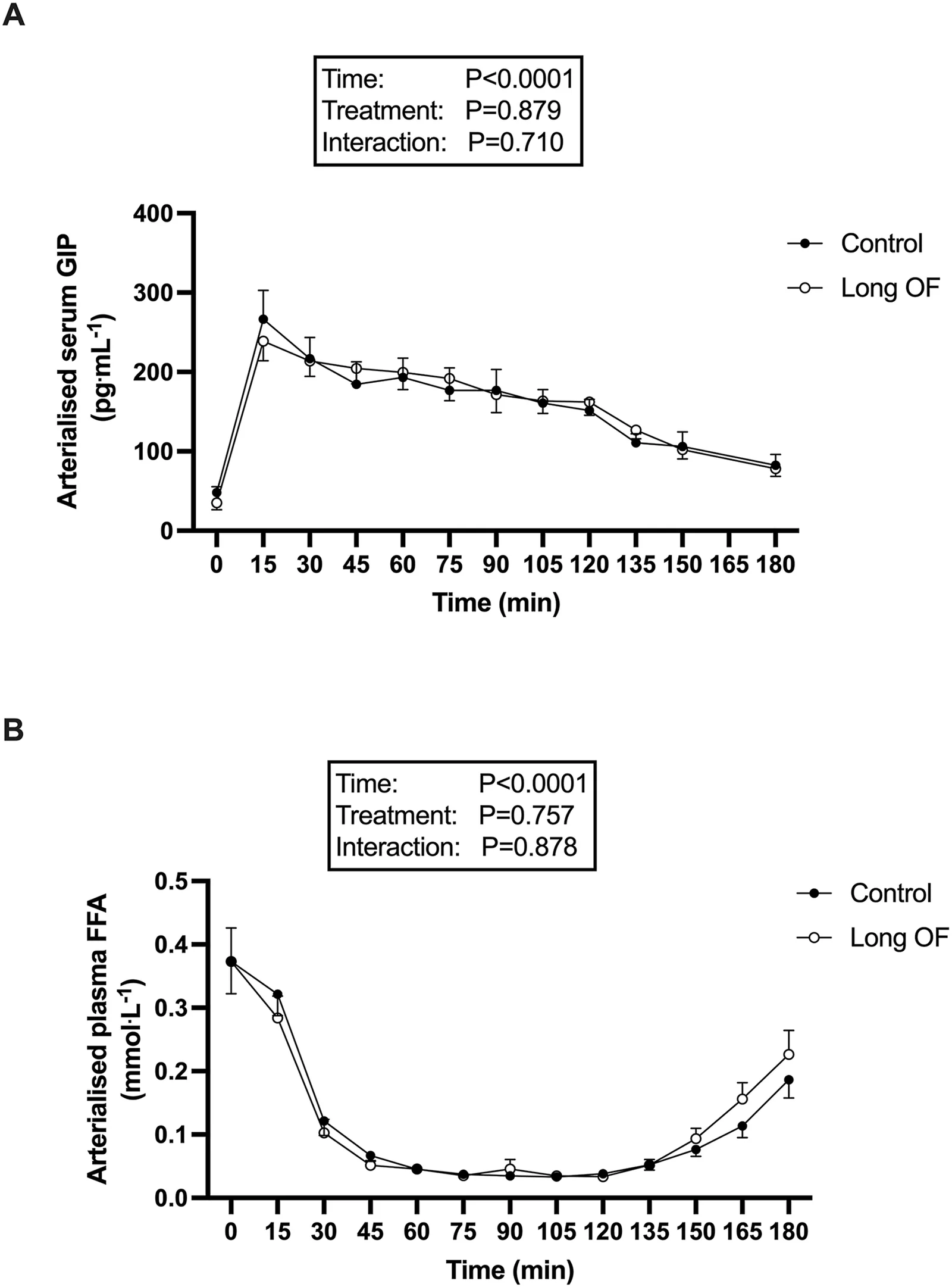
Serum GIP and plasma FFA responses. Effects of 16-h (Long OF) vs. 10-h (Control) overnight fast on (A) arterialized serum GIP concentrations; (B) arterialized plasma FFA concentrations. Values are means ± SEM; *n* = 9. *P* values for the main effects of 2-way analysis of variance are displayed in text boxes. FFA, free fatty acids; GIP, gastric inhibitory polypeptide; Long OF, prolonged overnight fast trial.

Fasting (baseline) serum GIP concentrations were lower in Long OF than Control [D (95% CI): −12.9 (−19.7, −6.2) pg/mL, *d*_*s*_ = 1.47, *P* = 0.002], but the postprandial response was similar between trials (interaction effect *P* = 0.710, Figure 5A). Fasting (baseline) plasma FFA concentrations and the extent of their postprandial suppression followed similar patterns between treatments (interaction effect *P* = 0.878, Figure 5B).

There were no significant differences in basal or postprandial respiratory exchange ratio, CHOox, and FATox between treatments (data not shown).

## Discussion

The main findings from this study show that a single bout of prolonged overnight fast does not acutely enhance skeletal muscle insulin sensitivity, although the postprandial serum insulin response was lower in Long OF than in Control. Furthermore, a prolonged overnight fast produced no significant effect on dietary protein digestion and amino acid absorption kinetics or postprandial incorporation or dietary protein-derived amino acids into muscle myofibrillar protein when compared with a standard overnight fast.

The role of overnight fasting duration on skeletal muscle anabolic sensitivity was investigated using intrinsically labeled milk protein to track dietary protein-derived amino acid incorporation into myofibrillar protein, alongside forearm BCAA uptake as an index for skeletal muscle anabolic sensitivity. Across all outcome measures (plasma amino acid enrichments, protein-derived amino acid incorporation in myofibrillar protein, and forearm BCAA uptake), a prolonged overnight fast produced no significant benefit over a standard overnight fast, with uniformly small effect sizes. These findings contrast with Jones et al. [4], who reported improved skeletal muscle BCAA uptake and insulin sensitivity after 2 wk of early TRE. The present results may suggest that the anabolic sensitivity improvements associated with chronic TRE represent a repeated-exposure adaptation rather than an acute consequence of fasting duration alone. However, our findings seem to align with those by Parr et al. [9], which used deuterated water (D_2_O) methodology to measure integrated MPS over a 10-d period in individuals with overweight/obesity and found no significant differences in daily myofibrillar protein synthesis rates between TRE and control, when dietary protein intake was matched. However, there is an important methodological distinction worth noting as D_2_O measures integrated MPS over days-to-weeks by capturing both the fasted and fed phases together. The intrinsically labeled milk protein approach used in the present trial allows far more granular resolution as it specifically tracks postprandial protein-derived amino acid incorporation into muscle protein in the first few hours after ingestion of a protein-rich meal, after a defined prolonged period of overnight fast. Therefore, the results from this study extend on previous findings by demonstrating that the acute postprandial response to a dietary-protein challenge is not negatively impacted by prolonged overnight fasting. When considering the findings by Parr et al. [9] alongside our own, extending an overnight fast to 16 h appears to have no detrimental effect on overnight MPS rates, which are typically lower than those observed in the morning after an overnight fast [[17], [18], [19], [20]]. Whether repeated overnight fast extension over weeks/months replicates chronic TRE benefits when postprandial MPS is measured directly remains to be established.

At first glance, the above findings appear to contradict previous evidence suggesting that TRE may lead to greater reductions in fat-free mass over prolonged periods of time when compared with an equivalent degree of dietary energy restriction achieved without fasting [6,7]. These findings are frequently interpreted as a loss of skeletal muscle mass resulting from a reduction in postprandial protein synthesis secondary to a reduction in daily energy and protein intake. However, this evidence is largely based on Dual-Energy X-ray Absorptiometry (DEXA)-derived fat-free mass losses, which may not translate into skeletal muscle losses per se, but rather reflect reductions in mass of other organs and/or the water compartment of lean mass. Therefore, further studies are required into the nutrient- and tissue-specific consequences of TRE paradigms to examine whether reductions in total and lean body mass when regularly experiencing extended periods of overnight fasting may be partly attributable to loss of skeletal muscle mass.

Another important finding from this study was a significant reduction in postprandial serum insulin concentrations in the Long OF trial, with insulin iAUC tending to be lower too. This occurred alongside equivalent blood glucose responses, a pattern typically interpreted as improved insulin sensitivity, whereby the same glycemic outcome is achieved with reduced prevailing serum insulin concentrations. However, forearm glucose uptake and the Matsuda Index were not significantly different between treatments in this study, suggesting that whole body and peripheral skeletal muscle glucose disposal were not acutely enhanced. Therefore, it can be argued that the reduction in postprandial insulin is an acute, early consequence of overnight fasting duration and likely precedes the longer-term TRE-induced improvements in insulin sensitivity and β-cells responsiveness previously observed in healthy individuals and those with prediabetes [4,5].

CGM data revealed a significant treatment by time interaction, characterized by higher diurnal glucose during Long OF, reflecting food intake compression into a shorter eating window, but attenuated nocturnal glucose responses. Despite this temporal redistribution, 24 h mean glucose and glycemic variability were unchanged. The attenuated nocturnal glucose aligns with findings on circadian insulin sensitivity, whereby β-cell responsiveness and insulin sensitivity were higher in the morning than in the evening [21]. This may explain the observation of nocturnal glucose intolerance after late dinner consumption [22], suggesting avoidance of late eating may partly explain the lower nocturnal glucose profile.

Another notable finding from this study was a lower fasting serum GIP response in Long OF compared with Control. GIP is a nutrient-sensitive incretin hormone secreted by duodenal K-cells [23], and its reduced fasting concentration after prolonged overnight fasting likely reflects diminished enteroendocrine stimulation during the extended nutrient-deprived period. Importantly, the postprandial GIP response was equivalent between interventions, indicating that K-cell secretory capacity to a meal was preserved, and that prolonged fasting selectively resets basal incretin tone rather than impairing dynamic meal-stimulated secretion. As GIP is a potent stimulator of pancreatic insulin secretion at basal and postprandial states [24], this finding may provide a plausible mechanistic link to the reduction in postprandial serum insulin response observed in Long OF. In support of this, in healthy individuals, acute GIP receptor antagonism during an oral glucose tolerance test reduced postprandial insulin secretion and increased blood glucose excursions [25], supporting the biological plausibility of this pathway. The present data also suggest that attenuation of basal incretin drive may be an early adaptation and contributing mechanism to reduced fasting insulin and improved insulin sensitivity after weeks of TRE, as previously observed [4,5].

In conclusion, a single bout of prolonged overnight fast in healthy individuals does not affect the postprandial incorporation of dietary protein-derived amino acids into muscle proteins, which may act as a protective mechanism limiting extensive muscle mass loss with extended periods of overnight fasting (a hallmark of TRE paradigms). However, a single episode of prolonged overnight fasting appears sufficient to reduce fasting GIP levels and postprandial insulin responses without inducing whole body or peripheral insulin sensitivity. These may be early adaptations contributing to improved insulin sensitivity and β-cell responsiveness after chronic TRE. Future work should examine whether susceptible populations, such as older adults or insulin-resistant individuals, show more pronounced responses.

## Authors contributions

The authors’ responsibilities were as follows – KT, RJ, JM: designed the study; KT: acquired funding (PhD studentship); JM, MK, AN, AS, RJ, PP, JMGS, LJCvL, KT: conducted the research; JM, MK, JMGS, KT: curated and analyzed the data and performed statistical analysis; KT, JM: wrote the original manuscript; JM, MK, AN, AS, RJ, PP, JMGS, LJCvL: reviewed and edited the manuscript; and all authors: read and approved the final manuscript before submission.

## Data availability

Data described in the manuscript, code book, and analytic code will be made available upon request.

## Declaration of Generative AI and AI-assisted technologies in the writing process

The authors declare that no generative AI or AI-assisted technologies were used in the writing of this manuscript.

## Funding

This research was supported by a BBSRC PhD studentship to RJ.

## Conflict of interest

LJCvL has received research grants, consulting fees, speaking honoraria, or a combination of these for research on the impact of exercise and nutrition on muscle metabolism. A full overview of research funding is provided at: https://www.maastrichtuniversity.nl/l.vanloon. All other authors report no conflicts of interest.
